# Identification of biallelic *POLA2* variants in two families with an autosomal recessive telomere biology disorder

**DOI:** 10.1038/s41431-024-01722-8

**Published:** 2024-11-30

**Authors:** Malin Kvarnung, Maria Pettersson, Pattra Chun-on, Maryam Rafati, Lisa J. McReynolds, Anna Norberg, Pedro Luis Moura, Ida Pesonen, Roza Chaireti, Boa Grönros Söderholm, Julia Burlin, Jenny Rydén, Eva Hellström Lindberg, Neelam Giri, Sharon A. Savage, Suneet Agarwal, Ann Nordgren, Bianca Tesi

**Affiliations:** 1 Department of Molecular Medicine and Surgery, Karolinska Institutet, Stockholm, Sweden; 2 Clinical Genetics and Genomics, Karolinska University Hospital, Stockholm, Sweden; 3 Division of Hematology/Oncology, Department of Pediatrics, Boston Children’s Hospital; Pediatric Oncology, Dana-Farber Cancer Institute, Harvard Medical School, Boston, MA USA; 4 Division of Cancer Epidemiology and Genetics, Clinical Genetics Branch, National Cancer Institute, Bethesda, MD USA; 5 Department of Medical Biosciences, Medical and Clinical Genetics, Umeå University, Umeå, Sweden; 6 Department of Medicine Huddinge, Center for Hematology and Regenerative Medicine, Karolinska Institutet, Stockholm, Sweden; 7 Department of Medicine Solna, Respiratory Medicine Unit, Karolinska Institutet, Stockholm, Sweden; 8 Department of Respiratory Medicine and Allergy, Karolinska University Hospital, Stockholm, Sweden; 9 Department of Hematology, Karolinska University Hospital, Stockholm, Sweden; 10 Department of Medicine Solna, Karolinska Institutet, Stockholm, Sweden; 11 Division of Nephrology, Danderyd University Hospital, Stockholm, Sweden; 12 Department of Clinical Genetics and Genomics, Sahlgrenska University Hospital, Gothenburg, Sweden; 13 Department of Laboratory Medicine, Institute of Biomedicine, Sahlgrenska Academy, University of Gothenburg, Gothenburg, Sweden; 14 Genomic Medicine Center Karolinska, Karolinska University Hospital, Stockholm, Sweden

**Keywords:** Cancer genetics, Ageing, Anaemia

## Abstract

*POLA2* encodes the accessory subunit of DNA polymerase α (polα)/primase, which is crucial for telomere C-strand fill-in. Incomplete fill-in of the C-rich telomeric strand after DNA replication has been proposed as a mechanism for Coats plus syndrome, a phenotype within the broader spectrum of telomere biology disorders (TBD). Coats plus syndrome has so far been associated with pathogenic variants in *POT1*, *CTC1*, and *STN1*. Here we report the findings of biallelic deleterious rare variants in *POLA2* gene detected by whole genome sequencing and segregation analysis in five young adults from two unrelated families. All five individuals displayed abnormally short telomeres and a clinical phenotype suggesting a TBD disorder with Coats plus features including retinal and gastrointestinal telangiectasias. Our results suggest *POLA2* as a novel autosomal recessive gene for a TBD with Coats plus features.

## Introduction

Telomere biology disorders (TBDs) constitute a heterogeneous group of genetic disorders caused by defects in telomere maintenance machinery. Associated with very short telomeres for age, TBDs are multisystem disorders characterized by a broad spectrum of manifestations including bone marrow failure, pulmonary fibrosis, and increased cancer risk [1, 2]. Individuals with TBDs due to biallelic variants in *CTC1*, *POT1*, and *STN1* genes usually display additional symptoms such as retinal and gastrointestinal (GI) telangiectasias and are referred to as having Coats plus [3, 4]. CTC1, STN1, and TEN1 are part of the CST complex and interacts with POT1 and TPP1. The CST complex, via the recruitment of the polymerase α (polα)/primase, a protein complex composed of POLA1, POLA2, PRIM1, and PRIM2, promotes telomere C-strand fill-in which controls the length of the 3’ G-overhang. Moreover, the polα/primase complex plays an essential role in the initiation of DNA synthesis during replication [5]. Functional studies suggest that Coats plus could result from incomplete fill-in of the C-rich telomeric strand after DNA replication [6]. Indeed, cells lacking CST–Polα-primase have been shown to lose 50–60 nt of telomeric CCCTAA repeats on the lagging strand per population doubling [7]. While mutations in two genes encoding the CST complex, *CTC1* and *STN1*, cause TBDs, mutations in genes encoding for Polα-primase have not yet been linked to human TBDs, although *PRIM1* and *POLA1* are linked to monogenic diseases.

Here we report genetic variants in *POLA2* and suggest its role as a novel autosomal recessive gene for a TBD with Coats plus features.

## Materials and methods

### Genetic investigations

Whole genome sequencing (WGS) was performed on DNA-isolated from peripheral blood on individuals AII:3 and BII:1. The data was analyzed using the previously described bioinformatic pipeline in clinical use for diagnostics of rare diseases at the Department of Clinical Genetics and Genomics of Karolinska University Hospital [8]. Human genome assembly hg19 was used as reference. Sanger sequencing was performed according to standardized protocols on blood-derived DNA to validate the findings from WGS data in the probands or carrier testing of siblings and parents. Breakpoint PCR using Platinum Taq DNA Polymerase kit (Invitrogen, Waltham, MA, USA) was performed on individuals from family B to confirm the rearrangement involving *POLA2*, followed by Sanger sequencing. Variants in *POLA2* were named according to reference transcript NM_002689.4 and reference genome hg19. Relative telomere length was estimated using quantitative polymerase chain reaction (qPCR) analysis from blood leukocytes as previously described [9]. In family A, telomere length was also measured by flow cytometry with in situ hybridization (flow-FISH) at Repeat Diagnostics, Inc (Vancouver, BC, Canada) [10]. Clinical and laboratory findings were retrieved from the medical charts.

### Molecular visualization and modeling of POLA2 missense variants

POLA2 domains were annotated according to the Uniprot entry of human POLA2 (UniProt accession ID Q14181). The already reported structure of POLA2 was downloaded from PDB, entry 8B9D, corresponding to the human replisome biological assembly reported by Jones ML et al. [11]. Due to a disordered region in the N-terminal domain spanning residues 112-167, the structure reported in PDB encompasses only residues 96-114 and 170-598, and is the only reported structure including residue 96. Therefore, to model the p.Ile96Thr variant, the structure of POLA2 predicted by AlphaFold 2.0 [12] was also investigated (AlphaFold ID: AF-Q14181). Both the p.Ile96Thr and p.Pro424Ala variants were generated in AlphaFold 2.0 through de novo sequence analysis with ColabFold [13]. All visualization and analysis steps were performed using UCSF Chimera v. 1.17.3 [14], including direct residue change and subsequent steps. Due to the internal location of Pro424, clash analysis was performed for a substitution predicted as benign by AlphaMissense (p.Pro424Ala) and the p.Pro424Leu, with clashing residues allowed to undergo 100 steps of steepest descent minimization. The AlphaFold-generated sequences spanning residues 168-598 of wildtype POLA2, p.Ile96Thr and p.Pro424Leu were then overlaid using the MatchMaker function of UCSF Chimera. Due to Ile96 being present at a potential protein-protein interaction site, the electrostatic potential at the surface of the changing residue was quantified and visualized in the AlphaFold wildtype POLA2 structure. Following this, the AlphaFold-generated sequences spanning the alpha-helix structure in residues 90-115 of wildtype POLA2, p.Ile96Thr were manually overlaid. Helix span distances were calculated between the core backbone atom of the first helix residue and last matching helix residue.

### Terminal restriction fragment analysis of *POLA2* genetic modifications in HEK293T cells

HEK293T (293 T) cells were obtained from ATCC, maintained in Dulbecco’s Modified Eagle Medium (Gibco) with 10% fetal bovine serum and were sub-cultured using 0.05% trypsin (Gibco) maintained at 37 °C in the presence of 5% CO2 in a humidified incubator.

Generation of human POLA2 p.Ile96Thr variant (chr11:65035030 T > C) knock-in clones was performed using CRISPR RNA (crRNA) and homology-directed repair (HDR) templates and a silent mutation to block the re-cutting increased the HDR accuracy. Both crRNA and HDR templates were designed from Integrated DNA Technologies (IDT) as described [15]. For generation of human *POLA2* knock-out clones including WT/WT/KO cells, we performed CRISPR/Cas9 using *POLA2* knock-out CRISPRevolution sgRNA EZ Kit (Synthego). The crRNA and HDR templates used to introduce the POLA2 p.Ile96Thr variant and sgRNA guides to knock-out *POLA2* are provided in Supplementary Table 1. For targeted CRISPR/Cas9 gene editing, 37 pmol Alt-R S.p. Cas9 Nuclease V3 (IDT) and 50 pmol of Alt-R crRNA (IDT) or 50 pmol chemically modified sgRNA (Synthego) were complexed at room temperature for 20 min. Then 200,000–400,000 293 T cells were combined with Cas9/gRNA complexes in 20 μl Buffer R (Thermo Fisher Scientific) with Alt-R Cas9 Electroporation Enhancer (IDT), followed by electroporation using the Neon Transfection System (Thermo Fisher Scientific) and the Neon Transfection 10 μl Kit. 293 T cells were electroporated with one pulse of 1200 V for 30 ms as described [16]. CRISPR gene-targeting validation was performed by Sanger sequencing on genomic DNA isolated from cell lines using PureLink Genomic DNA Mini Kit (Thermo Fisher Scientific). Primers used for POLA2 gene amplification and sequencing are provided in Supplementary Table 1. Sanger sequencing was performed by Genewiz and the efficiency of editing was analyzed by ICE software (Synthego). Next-generation amplicon sequencing was performed by the Massachusetts General Hospital DNA core facility and analyzed using CRISPResso2 software.

For Southern blot analysis of terminal restriction fragments (TRFs), genomic DNA was isolated from 293 T cells using the PureLink Genomic DNA Mini Kit (Invitrogen). Approximately, 1–3 μg gDNA was digested with RsaI (NEB) and HinfI (NEB) for 2–3 h at 37 °C and loaded onto a 0.6% agarose gel followed by Southern blotting onto Hybond-N+ membrane (Amersham). The membrane was probed using kit reagents as described [16]. Images were taken using Chemidoc Touch Imaging System (Bio-Rad). Quantification was performed using the WALTER webtool [17]. Statistical analysis was performed using GraphPad Prism version 10.2.0 (335).

## Results

Five individuals (two males and three females, age range: 31–40 years) from two unrelated Swedish families were studied. Family A (NCI-457) consisted of three affected siblings (AII:1, AII:2 and AII:3, Fig. 1A) while family B consisted of two affected siblings (BII:1 and BII:2, Fig. 1A). Neither family had a history of TBD-related diseases, however early graying of hair was reported for BI:1. The father (BI:2) in family B unexpectedly passed away due to a myocardial infarction at the age of 36 years. All five affected individuals presented symptoms consistent with a TBD, including macrocytic anemia with bone marrow hypocellularity (*n* = 5/5) managed with erythropoietin and blood transfusions, fibrotic interstitial lung disease or restrictive spirometric pattern (*n* = 4/5), graying of hair before age 20 (*n* = 4/5), and low-trauma fractures (*n* = 2/5). Moreover, symptoms consistent with Coats plus, such as exudative retinopathy (*n* = 5/5 bilateral in 4/5), cutaneous telangiectasias (*n* = 4/5), and GI bleeding due to angiodysplasia (*n* = 2/5) were noted. BII:1 was treated with tamoxifen for GI angiodysplasia with some effect. A brain MRI performed in BII:1 at age 29 revealed cysts and calcifications. All affected individuals in family A had moderate to severe kidney failure. Kidney biopsy performed on AII:1 and AII:3 showed glomerulosclerosis with mesangiolysis and glomerular microaneurysms. Both individuals who had undergone pregnancy (AII:1 and BII:2) experienced preeclampsia (AII:1: gestational week [GW] 29 and BII:2: GW26), a condition that has been described in up to 24% of pregnancies of women with TBDs [18]. Moreover, both affected individuals in family B were born during GW37 after induced labor due to maternal pre-eclampsia. Somatic investigations of bone marrow samples revealed a small clone with acquired activating *TERT* promoter mutations in two individuals. Three out of the five affected individuals are alive, to date; AII:2 died at age 31 due to complications from allogeneic hematopoietic stem cell transplantation, while BII:1 died at age 33 due to respiratory insufficiency. An overview of clinical manifestations in the five affected individuals is provided in Table 1.

**Fig. 1:**
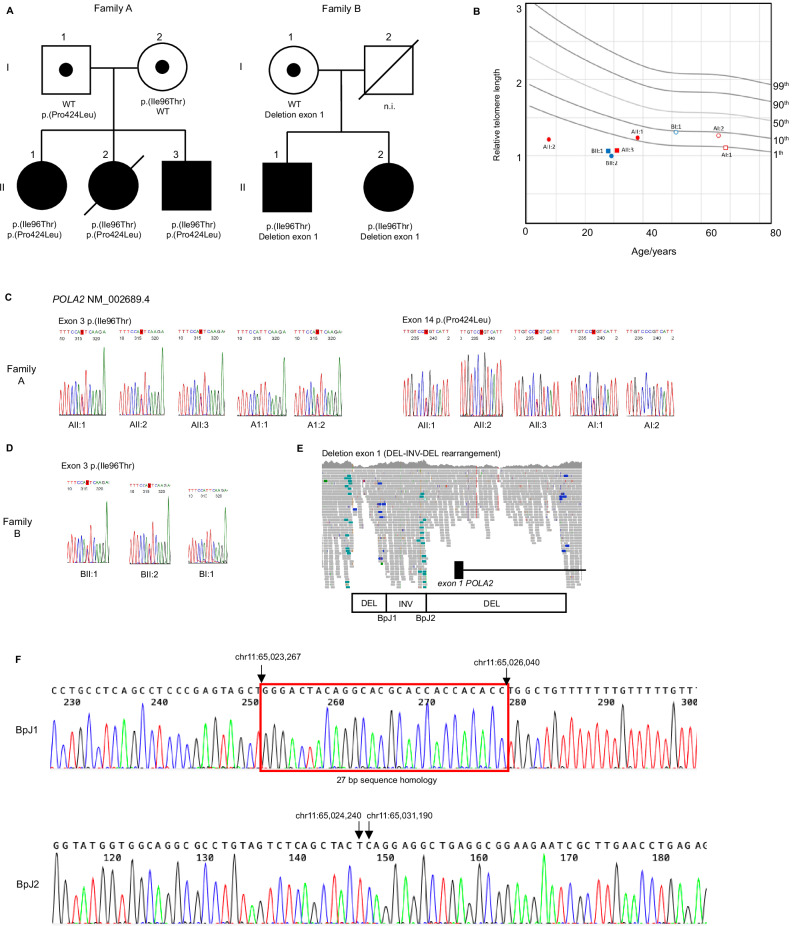
Overview of the included families and genetic findings. **A** Pedigrees of families A and B with three and two affected individuals, respectively. In family A the parents were confirmed to be heterozygous carriers of each *POLA2* variant, while in family B the mother was confirmed as heterozygous carrier of the complex rearrangement involving *POLA2*, and the father could not be tested as he was deceased. **B** Analysis of relative telomere length (RTL) by qPCR in peripheral blood leukocytes collected from family A (red symbols) and family B (blue symbols). Filled and unfilled symbols represent compound heterozygous and heterozygous genotypes, respectively. Squares and circles represent males and females, respectively. The reference percentiles were determined from telomere length analysis of blood leukocytes from 283 healthy subjects (0–78 years of age). The curves shown depict the first, 10th, 50th, 90th, and 99th normal percentiles at each age, where 50th represents the mean. **C** Sanger sequencing of *POLA2* (NM_002689.4) sequence variants performed on peripheral blood-derived DNA from all individuals tested in family A [c.287 T > C, p.(Ile96Thr) and c.1271 C > T, p.(Pro424Leu)] and (**D**) B [c.287 T > C, p.(Ile96Thr)] in family B. **E** Screenshot from Integrative Genome Viewer (IGV) over the complex rearrangement involving exon 1 of *POLA2* in family B. **F** Sanger sequencing of breakpoint junctions (BpJ) 1 and 2 of the complex rearrangement involving exon 1 of *POLA2* in BII:1. The HGVS nomenclature for the complex rearrangement is NC_000011.9:g.[65,023,295_65,024,239del;65,024,240_65,026,040inv;65,026,041_65,031,189del]]. The red box in BpJ1 highlights a 27-bp microhomology sequence found at both breakpoints. Arrows indicates specific genomic positions at the breakpoints.

**Table 1:** Overview of the clinical and laboratory features.

	Family A			Family B	
Patient	AII:1	AII:2	AII:3	BII:1	BII:2
Gender	F	F	M	M	F
Age (years)	40	Deceased at 31	32	Deceased at 33	31
Age (years) at onset	29	6 (Coats) DC Dx 29yo	Childhood (Coats) Other symptoms 30yo	3	4
First symptoms	Anemia and thrombocytopenia	Bilateral Coats exudative retinopathy; blind right eye	Coats exudative retinopathy left eye	Coats exudative retinopathy, blind right eye	Coats exudative retinopathy
Growth					
Adult length (cm)	160	157	180	188	173
Adult weight (kg)	59	50	80	61	54
BMI	23	19,5	24,7	**17,5**	18,7
Hematological					
Hemoglobin (g/L) Ref female: 117-153 Ref male: 134-170	**111** (29 yo) → **85** **75** lowest during pregnancy **79** lowest non-pregnant	**98** (21yo) → **64** (lowest, 31yo)	132 (21 yo) → **88** Blood transfusion twice age 32	**112** (28 yo) → **77**	132 (20 yo) → **89**
MCV (fl) Ref: 82–98	**97–115**	**106**	**100-106**	**103-110**	**100-103**
Platelet count (x10^9/L) Ref female: 165-387 Ref male: 145-348	**72**-170 (lowest during pregnancy); 124 lowest non-pregnant	**33** (lowest 31yo)	221 – 249, relatively stable	**98**-258	198 (20 yo) → **150** (32 yo)
WBC count (x10^9/L) Ref: 3,5–8	**2,5**-7	**1,8** (lowest)	**2,8** – 5,7	5,2-6,4	3,6-5,6
Bone marrow cellularity	50% at 29 yo 30% at 39 yo	20-40% at 29yo <5% at 31yo	50% at 30 yo 40% at 32 yo (megaloblastoid features)	40% at 29 yo	30% at 26 yo
Bone marrow cytogenetics	46,XX	46,XX	46,XY	46,XY	46,XX,del(7)(q2?2q3?)[4]/46,XX[6] at 26 yo
Bone marrow NGS panel	Normal	n.a.	Normal	Normal	Normal
Bone marrow TERT promoter	*TERT* promoter c.-124C > T VAF 2%	n.a.	n.a.	n.a.	*TERT* promoter c.-57A > C VAF 4% (27 yo) > 13% (30 yo)
Retinal telangiectasias/exudates (Coats disease)	Y, bilateral temporal peripheral non perfusion	Y, bilateral	Y, unilateral	Y. bilateral	Y. bilateral
Skin telangiectasias	Y	Y	N	Y	Y
GI-bleeding	N	Y, gastric antral vascular ectasia	N	Y, gastric antral vascular ectasia	N
Premature graying of hair	Y	Y	N	Y, since 15 yo	Y, since 13 yo
Mucocutaneous triad	Lacy, reticular pigmentation upper trunk	None	Dysplastic nails	Dysplastic nails	Lacy, reticular pigmentation upper trunk
Osteoporosis	n.a.	Not known	n.a.	Y	N, osteopenia
Fractures	N	None reported; R clavicle osteitis	Y, traumatic x2	Y, multiple low-trauma fractures	Y, multiple low-trauma fractures
Pulmonary disease	N	Y, apical fibrosis; mildly reduced DLCO	Y, fibrotic ILD (32 yo)	Y, fibrotic ILD	Y, restrictive pattern on spirometry (26 yo)
Elevated liver enzymes	N	N; fibrosis on imaging; ascites	N	**N**	N
ESR (mm)	**49-77**	n.a.	**97-140**	**41-63**	**42**
Creatinine (micromol/L)	65 → **181** (stable increase)	**114** (29yo) → **190** (31yo)	72 (21 yo) → **184** (32 yo)	80	52
eGFR (Creatinine) mL/min/1.7	85 (age 32) → **29** (age 40)	**64** (29yo) → **42** (31yo)	80 (26 yo) → **35** (32 yo)	>90	>90
Urine albumin/creatinine ratio (mg/mmol)	15 → **70**	**53**	30 → **99**	n.a.	n.a.
Kidney biopsy	Glomerulopathy with segmental sclerosis (50%), mesangiolysis and glomerular microaneurysms		Glomerulopathy with segmental sclerosis (20%), mesangiolysis and glomerular microaneurysms	n.a.	n.a.
Preeclampsia	Y	n.a.	n.a.	n.a.	Y
Brain MRI	n.a.	Normal at 29yo	n.a.	Cysts and calcifications at 29 yo	n.a.
Treatment	EPO (40yo) Enalapril	HCT; matched unrelated donor; alemtuzumab/fludarabine conditioning	Blood transfusion prn age 32 (4 units) EPO Cardesartan	Blood transfusion prn Tamoxifen Iron supplement Since age 33 TPN	EPO Blood transfusion prn

Range for laboratory values is indicated as “-“ ; while trend over time is indicated as “ →”. Values outside reference internals are indicated in bold.

*Yo* year old, *n.a.* not applicable/available, *Y* yes, *N* no. Other abbreviations used in the table are *DC* dyskeratosis congenita, *Dx* diagnosis, *EPO* erythropoietin, *ESR* erythrocyte sedimentation rate, *GFR* glomerular filtration rate, *GI* gastrointestinal, *gw* gestational week, *HCT* hematopoietic cell transplantation, *ILD* interstitial lung disease, *NGS* next-generation sequencing, *PRN* pro re nata, *VAF* variant allele frequency, *WBC* white blood cells.

Relative telomere length was estimated using quantitative polymerase chain reaction (qPCR) analysis from blood leukocytes and showed reduced telomeres in all affected individuals (Fig. 1B). Short telomeres were confirmed by flow-FISH in family A (Supplementary Fig. 1).

Overall, family history and clinical presentation were suggestive of an autosomal recessive TBD. WGS performed on AII:3 and BII:1 did not reveal any disease-causing variants in known genes for TBDs (Supplementary Table 2). Further filtering for rare deleterious variants detected two heterozygous missense variants in the *POLA2* (NM_002689.4) gene, c.287 T > C, p.(Ile96Thr) and c.1271 C > T, p.(Pro424Leu) in patient AII:3. The same missense variant p.(Ile96Thr) was also identified in heterozygous state in BII:1, together with a heterozygous intragenic structural variant (deletion-inversion-deletion, 7.8 kb in total) of *POLA2*, resulting in deletion of the *POLA2* 5’ terminus and exon 1 (NC_000011.9:g.[65,023,295_65,024,239del;65,024,240_65,026,040inv;65,026,041_65,031,189del]).

**Fig. 2:**
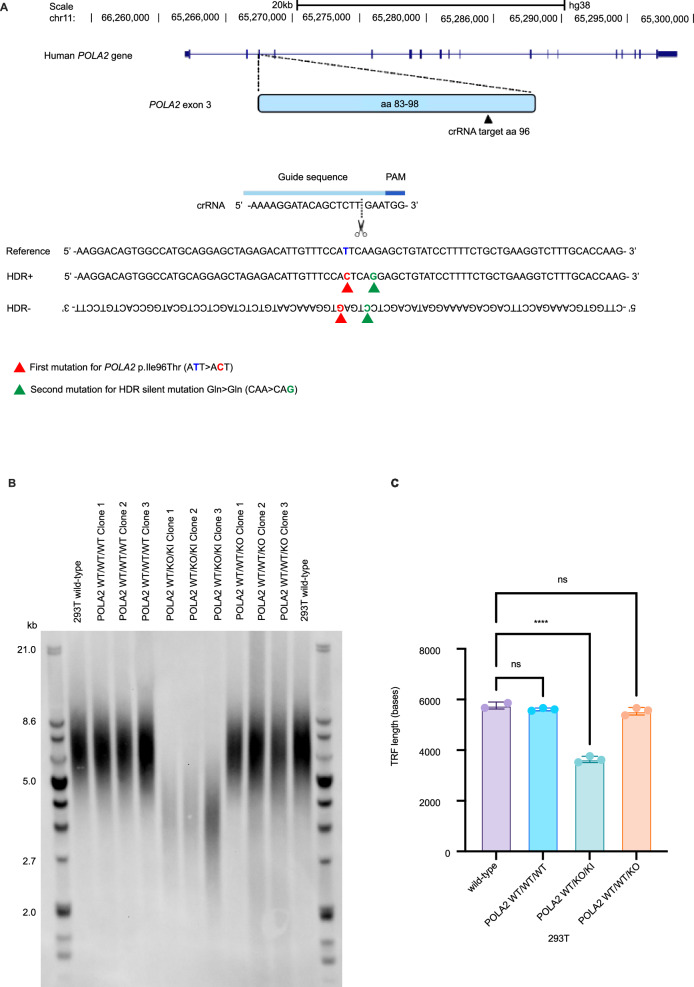
Functional assessment of p.Ile96Thr in 293 T cells. **A** Schematic of the Cas9/crRNA-targeting sites in human *POLA2* locus on exon 3 at amino acid 96. Exons are shown as blue boxes. The crRNA-targeting sequence is labeled as Guide sequence, and the protospacer-adjacent motif (PAM) sequence is labeled as PAM. The homology arms specific to the gene target are flanking the nucleotide to be changed (Ile>Thr or ATT > ACT on +strand labeled in red) and HDR silent mutation (Gln>Gln or CAA > CAG on +strand labeled in green). **B** Terminal Restriction Fragment Southern blot showing telomere lengths four weeks after CRISPR/Cas9 genetic modification of the triploid *POLA2* locus in 293 T cells, indicating wild-type (WT), monoallelic knock-out (KO), and p.Ile96Thr knock-in (KI) alleles. WT/WT/WT represents clones with no editing of the loci (KI score 0–1%); WT/KO/KI represents *POLA2* p.Ile96Thr on one allele (KI score of 24–35%), and one disrupted allele (KO); and WT/WT/KO indicates *POLA2* disruption on one allele (KO score of 28–35%). **C** Quantitation of telomere length in (**B**). Statistical analysis was done by one-way ANOVA followed by Dunnett’s multiple-comparisons test. ****P < 0.0001, ns: not significant.

Segregation analysis performed in both families confirmed compound heterozygosity for the *POLA2* variants in all affected individuals (Fig. 1C–F). The *POLA2* missense variant p.(Ile96Thr), shared between the two families, was detected in a heterozygous state in 12 additional individuals in a local clinical database of 11,002 individuals who underwent clinical exome or genome sequencing at Karolinska University Hospital. The variant is also present in a heterozygous state in 51 individuals in the gnomAD database (minor allele frequency [MAF]: 0.003181%; gnomAD v.4). Based on data from gnomAD v.2.1.1, where Swedish ancestry is specified, this variant was present in 22 individuals of which 18 of them were of Swedish origin. The MAF for this variant in gnomAD v.2.1.1 was therefore 0.00887% for the whole cohort and 0.06899% in the Swedish population. The other missense variant, p.(Pro424Leu) has ten heterozygous carriers in the gnomAD database v4 (MAF 0.0006841%). Intragenic structural variants were rare in gnomAD v.4 and no identical event to the one identified in BII:1 was found in gnomAD nor in our local clinical database. The identified missense variants are located in different domains of POLA2, yet both show conservation across species (Supplementary Fig. 2A–C). Both variants are predicted as deleterious by in-silico prediction tools (Supplementary Table 3). The variant p.(Ile96Thr) is located in the N-terminal domain of POLA2 which has been proposed to represent a site of protein-protein interaction, potentially with CTC1 [19]. No large differences were noted when modeling the interaction surface with AlphaFold (Supplementary Fig. 2F). However, overlay of AlphaFold-generated models for p.(Ile96Thr) revealed a shortened alpha-helix compared to wild-type POLA2 (Supplementary Fig. 2G). The variant p.(Pro424Leu) is instead located in an inner core of the protein; clash modeling suggests a steric clash is introduced by p.(Pro424Leu) compared to a hypothetical p.(Pro424Ala) substitution that is predicted benign by AlphaMissense (Supplementary Fig. 2D, E). The missense variants were not classified according to the guidelines from the American College of Medical Genetics and Genomics, as these guidelines are not intended to fulfill the needs of the research community in its effort to identify new disease-causing genes [20]. We thereafter asked whether the POLA2 p.(Ile96Thr) variant could impact telomere length maintenance in human cells using CRISPR/Cas9 genome engineering in 293 T cells. Guide RNAs were designed targeting the human *POLA2* locus on exon 3 at amino acid 96, complexed with Cas9, and electroporated alongside HDR templates into 293 T cells (Fig. 2A). Electroporated cells were subcloned and genotyped. We successfully engineered three subclones with knock-in of p.(Ile96Thr) in *POLA2* exon 3, at a frequency likely reflective of HDR at one of three loci in this triploid cell line as determined by amplicon sequencing. Of note, in all three subclones, one allele remained wild-type and the third allele was disrupted by out-of-frame insertion/deletion (WT/KO/KI). Three subclones with no alterations at *POLA2* exon 3 (WT/WT/WT) and three subclones with disrupting mutations on one allele (WT/WT/KO) were used as controls. We examined telomere length in all subclones by TRF Southern blot four weeks after engineering. We found that all three subclones carrying the *POLA2* p.(Ile96Thr) mutation showed significant telomere shortening with a median length of ~4 kb, compared to wild-type subclones (WT/WT/WT) showing a median length of ~6 kb, similar to unmanipulated 293 T cells and WT/WT/KO controls (Fig. 2B, C).

## Discussion

We identified biallelic deleterious variants in the accessory subunit of DNA polymerase alpha 2 (*POLA2*) gene in five individuals with a TBD from two unrelated families. Our study is first to suggest *POLA2* as a novel autosomal recessive gene for TBD.

The *POLA2* gene encodes the regulatory subunit of the RNA primer synthesizing complex polα/primase, which is crucial for the extension of the telomere C-strand [7, 11]. Moreover, the polymerase-alpha-primase complex plays an essential role in the initiation of DNA synthesis during replication [5], and POLA2 has been shown to also have a role in double strand breaks repair [21]. There are known human disorders described for four genes that closely interact with POLA2 as part of the CST–Polα-primase. Pathogenic variants in genes *POLA1* and *PRIM1*, respectively, cause severe pediatric multisystem disorders with growth restriction (MIM# 301220, 301030 (*POLA1*) and MIM# 620005 (*PRIM1*)), while pathogenic variants in the CST-complex genes *CTC1* and *STN1* cause TBDs with phenotypes consistent with Coats plus.

The phenotypes of the affected individuals resemble those associated with pathogenic variants in CST-complex genes *CTC1* and *STN1* as well as of patients with pathogenic variants in *POT1* [6]. All affected individuals had abnormally short telomeres. Interestingly, tested parents (*n* = 3) were found to have telomere lengths under the 10th percentile compared with age-matched controls, similar to heterozygous carriers of autosomal recessive TBDs [1]. The detection of somatic clones with activating variants in *TERT* promoter in bone marrow samples, which likely represents events of somatic genetic rescue previously reported in TBDs [1], further argues for this being a new bona fide TBD. Renal failure, observed in Family A only, is a very rare complication of TBD [22]. Due to the roles of *POLA2* beyond telomere maintenance, it is possible that pathogenic variants in this gene may cause a broader phenotype than what was previously observed in TBDs. However, even though screening for genetic variants with known association to renal disorders did not identify any pathogenic variant, we cannot completely exclude the presence of a second genetic aberration, which alone or in conjunction with the *POLA2* variants, could have contributed to the renal phenotype. Further delineation of *POLA2*-related phenotypes in additional families is needed to understand the potential causative link between POLA2 and kidney disease.

In total, three deleterious variants in *POLA2* were detected in two different families. One of the variants p.(Ile96Thr) was shared between the families and may constitute a founder variant in the Swedish population. This variant is located at the N-terminal of POLA2, a region that has been proposed to interact with CTC1 [19]. Knock-in experiments in 293 T cells revealed significantly reduced telomere length in TRF assay in clones carrying the p.(Ile96Thr) allele compared to wild-type, which could reflect either a dominant negative effect or partial loss of function with respect to telomere maintenance functions. We hypothesize that the complex rearrangement involving exon 1 identify in family B represents a loss of function variant, while the p.(Pro424Leu) is predicted to cause steric clash on the structure of POLA2. Future mechanistic studies are needed to elucidate if pathogenic variants in human *POLA2* affect only telomere length maintenance or also genome replication and DNA repair.

In summary, we report five individuals from two families with biallelic deleterious variants in *POLA2*, short telomeres, and a phenotype which is similar to those linked to mutations affecting the CST-complex. Our results suggest *POLA2* as a novel autosomal recessive gene for a TBD with Coats plus features.

## Supplementary information

Supplementary table 1

Supplementary table 2 and 3

Supplementary figure legends

Supplementary figure 1

Supplementary figure 2

## Data Availability

*POLA2* variants were submitted to ClinVar (Accession numbers: SCV004229047, SCV004228467, SCV004228468). The ethical approval does not permit sharing of WGS data. Access to de-identified data that are not provided may be requested via the corresponding author.
