# Supplementary material for: Identification of biallelic POLA2 variants in two families with an autosomal recessive telomere biology disorder
Source: Eur J Hum Genet. 2024 Nov 30;33(5):580–7. doi: 10.1038/s41431-024-01722-8 (PMC12048608; doi:10.1038/s41431-024-01722-8)
Supplement: Supplementary file 1 — Supplementary table 1 [file 41431_2024_1722_MOESM1_ESM.docx]

Supplementary table 1

| **Chemically modified crRNA and HDR templates were used to introduce the POLA2 p.Ile96Thr variant** | |
| --- | --- |
| Alt-R crRNA – POLA2 target exon 3 for p.Ile96Thr Knock-in  crRNA | 5’-AAAAGGAUACAGCUCUUGAA-3’ |
| POLA2 p.Ile96Thr HDR plus strand (HDR+) | 5’-AAGGACAGTGGCCATGCAGGAGCTAGAGACATTGTTTCCACTCAGGAGCT  GTATCCTTTTCTGCTGAAGGTCTTTGCACCAAG- 3’ |
| POLA2 p.Ile96Thr HDR minus strand (HDR-) | 5’-CTTGGTGCAAAGACCTTCAGCAGAAAAGGATACAGCTCCTGAGTGGAAAC  AATGTCTCTAGCTCCTGCATGGCCACTGTCCTT- 3’ |
| sgRNA POLA2 Knock-out guide 1 | 5’ -GGAUGUCCAGAGGCACUAAC- 3’ |
| sgRNA POLA2 Knock-out guide 2 | 5’ -CGUGAGUUGUAUUUCUGGGA- 3’ |
| sgRNA POLA2 Knock-out guide 3 | 5’ -CACAGGGAGUAUCUUGGUCU- 3’ |
| **Primers sequences used for POLA2 amplification and sequencing** | |
| POLA2 exon3 FWD | 5’-CTTTTGAAAACACCTCTGCTATT-3’ |
| POLA2 exon3 REV | 5’-TGTTGCCCAGGTTGGTCAC-3’ |
| POLA2 exon3 SEQ | 5’-ATCTTTTCTTTTTCAGTTTCTGAGCAA-3’ |
| POLA2 KO FWD | 5’ -AGGTCTGGGTATGTCCAACC- 3’ |
| POLA2 KO REV | 5’ -GGGCTTCCATGAGGACATCT- 3’ |
| POLA2 KO SEQ | 5’ -TCCAACCCCATTAAACTGATTCAATTTATA- 3’ |
