# Supplementary material for: Identification of biallelic POLA2 variants in two families with an autosomal recessive telomere biology disorder
Source: Eur J Hum Genet. 2024 Nov 30;33(5):580–7. doi: 10.1038/s41431-024-01722-8 (PMC12048608; doi:10.1038/s41431-024-01722-8)
Supplement: Supplementary file 2 — Supplementary table 2 and 3 [file 41431_2024_1722_MOESM2_ESM.docx]

| **Symbol (HGNC)** |
| --- |
| *ACD* |
| *CTC1* |
| *DKC1* |
| *NAF1* |
| *NHP2* |
| *NOP10* |
| *PARN* |
| *POT1* |
| *RPA1* |
| *RTEL1* |
| *STN1* |
| *TERC* |
| *TERT* |
| *TINF2* |
| *WRAP53* |
| *ZCCHC8* |

**Supplementary table 2. List of known TBD genes analyzed in the probands**

| **Chr** | **Start** | **End** | **Ref** | **Alt** | **HGVS c.** | **HGVS p.** | **Polyphen-2 (score)** | **SIFT (score)** | **REVEL** | **CADD-PHRED** | **AlphaMissense** |
| --- | --- | --- | --- | --- | --- | --- | --- | --- | --- | --- | --- |
| 11 | 65035030 | 65035030 | T | C | c.287T>C | p.(Ile96Thr) | probably damaging (0.922) | deleterious (0.01) | 0.302 | 25.2 | 0.66 (pathogenic) |
| 11 | 65061650 | 65061650 | C | T | c.1271C>T | p.(Pro424Leu) | probably damaging (1) | deleterious (0.01) | 0.80 | 32 | 0.81 (pathogenic) |

**Supplementary table 3 – In-silico prediction for the identified *POLA2* missense variants**
