# Supplementary material for: Identification of biallelic POLA2 variants in two families with an autosomal recessive telomere biology disorder
Source: Eur J Hum Genet. 2024 Nov 30;33(5):580–7. doi: 10.1038/s41431-024-01722-8 (PMC12048608; doi:10.1038/s41431-024-01722-8)
Supplement: Supplementary file 3 — Supplementary figure legends [file 41431_2024_1722_MOESM3_ESM.docx]

**Supplementary figure legends**

**Supplementary figure 1.** Lymphocyte telomere lengths measured by flow cytometry with *in situ* hybridization (flow FISH) at Repeat Diagnostics (Vancouver, Canada). Percentiles were derived from more than 400 healthy control individuals as reported in Alter et al, *Blood* 2007;110(5):1439—47.

**Supplementary figure 2.** **A)** Lollipop plot indicating the location and amino acid residue changes of the missense variants of *POLA2* (DNA Polymerase Alpha Subunit B) reported in this study. **B)** Multiple protein sequence alignment of POLA2 of different species. POLA2 orthologs were obtatined through UCSC Genome Browser. **C)** Overview of *POLA2* structure and interacting partners based on the human replisome biological assembly (PDB: 8B9D; PMID: 37506699). Structures without a defined direct interaction with *POLA2* (min. atomic distance < 5 Å) were omitted in this representation of the original model. The Ile96 and Pro424 residues are respectively highlighted in boxes 1 and 2. **D)** Clash modelling of the predicted benign variant p.Pro424Ala (left panel) and the predicted pathogenic variant reported in this study p.Pro424Leu (baseline clashes in middle panel; clashes after 100 steps of structure minimisation in right panel). Clashing atoms are represented in red and counted below each panel. **E)** Matched overlay of AlphaFold-generated models for the wildtype *POLA2* structure (white), p.Ile96Thr variant (grey) and p.Pro424Leu variant (yellow). The knock-on change in beta strand conformation associated with p.Pro424Leu is highlighted in the residues F455 (original beta strand endpoint) and V456 (new beta strand endpoint). **F)** Surface visualization of the AlphaFold wildtype *POLA2* model (left), including electrostatic potential colouring (Coulombic colouring) for the wildtype I96 residue (middle) and the p.Ile96Thr variant (right). **G)** Artificial overlay of AlphaFold-generated models for the wildtype *POLA2* structure (white) and p.Pro424Leu variant (grey), compared with the p.Ile96Thr variant (yellow). Alpha-helix lengths are quantified for both the wildtype and mutant sequences.
