# Supplementary material for: Identification of biallelic POLA2 variants in two families with an autosomal recessive telomere biology disorder
Source: Eur J Hum Genet. 2024 Nov 30;33(5):580–7. doi: 10.1038/s41431-024-01722-8 (PMC12048608; doi:10.1038/s41431-024-01722-8)
Supplement: Supplementary file 4 — Supplementary figure 1 [file 41431_2024_1722_MOESM4_ESM.pdf]

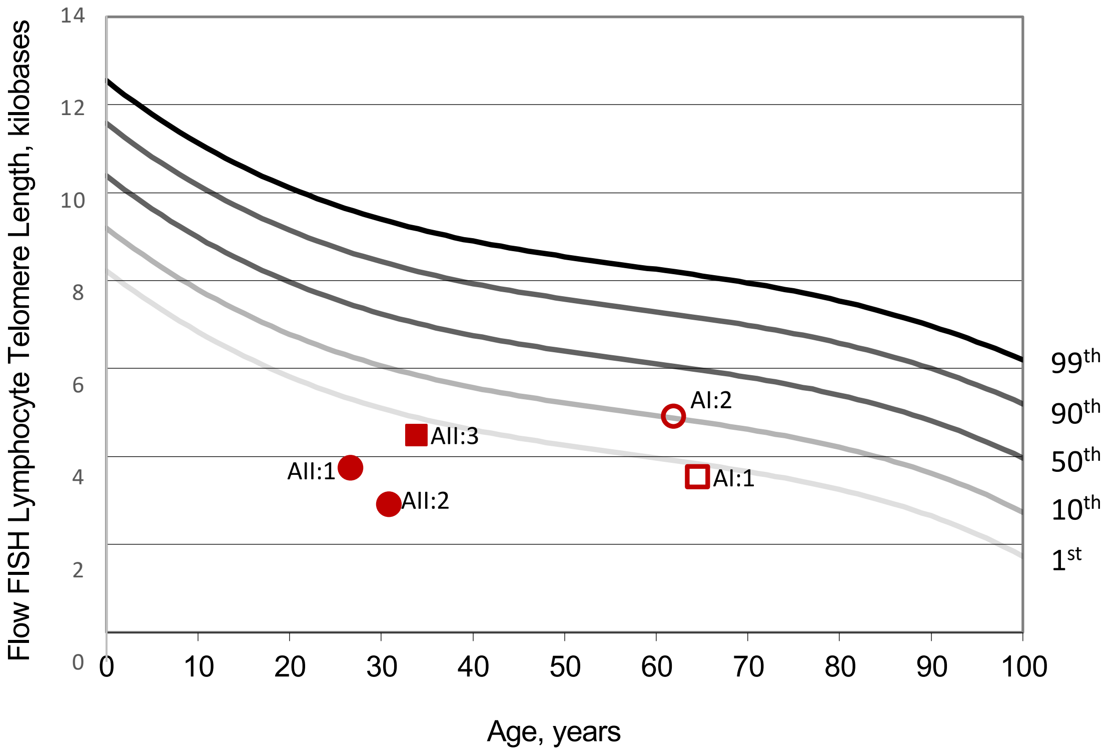

Supplementary Figure 1: Lymphocyte telomere lengths measured by flow cytometry with *in situ* hybridization (flow FISH) at Repeat Diagnostics (Vancouver, Canada). Percentiles were derived from more than 400 healthy control individuals as reported in Alter et al, *Blood* 2007;110(5):1439—47.
