# Supplementary figures and images for: Identification of biallelic POLA2 variants in two families with an autosomal recessive telomere biology disorder
Source: Eur J Hum Genet. 2024 Nov 30;33(5):580–7. doi: 10.1038/s41431-024-01722-8 (PMC12048608; doi:10.1038/s41431-024-01722-8)

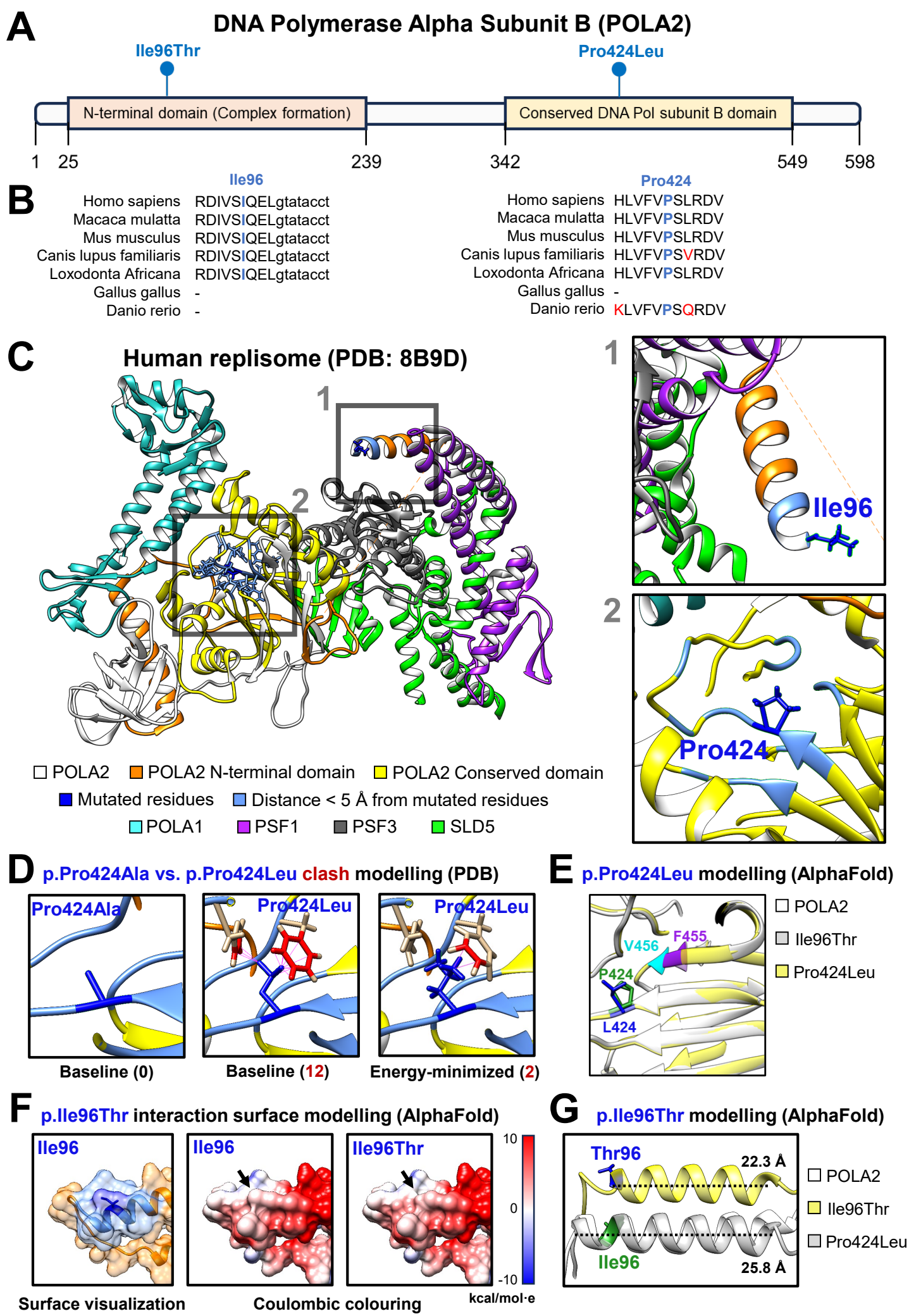

Supplement: Supplementary file 5 — Supplementary figure 2 [file 41431_2024_1722_MOESM5_ESM.pdf]
